# Mental and physical comorbidity in an Arab primary health care setting

**DOI:** 10.1186/s12888-018-1903-8

**Published:** 2018-09-27

**Authors:** Sulaiman Alkhadhari, Aseel Omran Alsabrrie, Jude Uzoma Ohaeri, Ramani Varghese, Muhammad Ajmal Zahid, Benoit H Mulsant

**Affiliations:** 1 Department of Psychiatry, Faculty of Medicine, Safat, Kuwait; 2 Abdullah & Shereefa Almehry Health Center (Alkhaldiya Polyclinic), Kuwait City, Kuwait; 3 Department of Psychiatry, University of Toronto, Toronto, Canada; 4 Department of Psychiatry, Faculty of Medicine, Health Sciences Center, PO Box 24923, 13110 Safat, Kuwait

**Keywords:** Primary care attenders, Mental health, Depression, Anxiety, Physical illness, Prevalence, Comorbidity

## Abstract

**Background:**

There are no published studies on the comorbidity of common mental conditions (anxiety, depression, and somatization) and physical illnesses in the Arab world. Our aim was to estimate the prevalence of common mental conditions comorbid with physical illnesses among primary care attenders in Kuwait, and the sociodemographic characteristics associated with this comorbidity.

**Methods:**

The Patient Health Questionnaires for somatization, anxiety and depression (PHQ-SAD) were administered to a representative sample of 1046 attenders (M: F = 429: 617; mean age 37.6, SD 12.7) seen in primary care clinics in Kuwait. Based on well-established cut-off scores, the presence and severity of three mental conditions –depression, anxiety, and somatization—was ascertained; physical diagnoses were ascertained by the attending physicians.

**Results:**

Of 1046 respondents, 442 (42.3%) had at least one mental condition and 670 (64.1%) had a physical illness diagnosis, viz.: diabetes mellitus (248/670 = 37.0%), hypertension (229/670 = 34.2%), asthma (82/670 = 12.2%), non-chronic physical illnesses (63/670 = 9.4%), or heart disease (48/670 = 7.2%), with 34.4% (360/1046) having mental-physical comorbidity. Male: female ratio for the 670 subjects was 287: 383. The unadjusted odds ratio (OR) for having a mental condition in those with a physical illness vs. those without a physical illness was 4.16 (95% C.I. = 3.12, 5.55). Comorbidity was associated with older age, being divorced or widowed, a lower level of education, and poorer living conditions. Regardless of the physical illness, the most frequent comorbid mental disorder was somatization. The prevalence and severity of mental conditions were associated with the number of physical illnesses.

**Conclusion:**

As has been reported in other parts of the world, somatization, anxiety, and depression are highly prevalent among primary care attenders in Kuwait and they are typically comorbid with physical illness. Strategies for their prevention and treatment need to take into consideration their association with physical illness and social disadvantage.

## Background

The literature has consistently shown that anxiety, depression, and somatization are highly prevalent among primary care attenders. The estimated prevalence rates in recent large international studies are: 31–35.8% for depression, 19–25.6% for anxiety, and 18–28.8% for somatoform disorders [1, 2]. The studies indicated that these are the commonest mental disorders presenting in primary care settings.

This high prevalence has been attributed in large part to their co-occurrence with the physical illnesses that are known to be commonly prevalent in primary care settings, such as diabetes mellitus, hypertension, heart diseases and asthma [3]. The issues surrounding comorbidity of mental and physical disorders have been of international concern, being the subject of a book review by WHO consultants [3]. A major concern is that the successes of medicine prolong life for subjects with these physical illnesses, thus adding years at risk for mental comorbidity [3]. The pathways linking comorbid mental and physical conditions are complex and bidirectional, such that, a physical disorder may cause a mental one, a mental disorder may place a person at risk for a physical one, and some mental and physical disorders share risk factors, such as chronic stressful social life situations, physical inactivity, overweight, smoking, substance use disorders and endocrine dysregulation [3]. Regardless of the chronological order in which they occur, or the causal pathways linking them, the comorbidity of a mental and physical condition may impact the mode of presentation, clinical severity, response to treatment, and burden of illness of both conditions [1–4]. Despite the robustness of these findings and the availability of effective interventions that integrate the care of common mental and physical conditions [5], many physicians in the Arab world pay scant attention to comorbidity issues [6–8], where the high prevalence and co-occurrence of anxiety and depression has been reported in both general population samples [9, 10] and primary care settings [7, 8]. However, to our knowledge, there are no published studies of the comorbidity of these common mental conditions and physical illnesses in the Arab world.

### Objectives

Thus we conducted a study in a representative sample of Kuwaiti primary care attenders, the objectives of which were: to assess the comorbidity of depression, anxiety, and somatization with common chronic physical illnesses - diabetes mellitus, hypertension, heart disease, and asthma, as well as non-chronic medical conditions; and to explore the patients’ characteristics associated with this comorbidity.

## Methods

The methods for sampling and assessing participants have been described in detail elsewhere [6] and are briefly summarized below.

### Sampling

After the ethics approval for the study was obtained from the Scientific and Research Committee, Ministry of Health, Kuwait, 16 of 87 primary health centers in Kuwait were randomly selected. A sample size of 1000 participants was estimated to provide a precision of 2%, given the estimated 16% prevalence of psychiatric disorders among PHC attenders in routine consultation, and a significance level set at 0.05. Thus, 1046 participants were recruited to allow for up to 5% cases with missing data. Participants gave written informed consent.

### Physical diagnoses

Physical diagnoses were ascertained by the primary care physicians based on the International Classification of Diseases, 10th edition (ICD-10) [11]. When necessary, these diagnoses were confirmed by further investigations, (such as radiological and electro-encephalograhic) or hospital-based consultants.

### Psychopathology instruments

The Patient Health Questionnaire-Somatic/Anxiety/Depression (PHQ-SAD) is an easy-to administer brief assessment tool, designed to measure probable rates of psychiatric morbidity in the primary care settings, based on the DSM-IV [12]. The PHQ-SAD has been widely used to assess the presence and severity of depression, anxiety, or somatization and it has been reported to have good psychometric properties [13–16]. It comprises three questionnaires: the Patient Health Questionnare-9 (PHQ-9); the Generalized Anxiety Disorder-7 (GAD-7), and the Patient Health Questionnaire-15 Somatic Symptoms (PHQ-15).

The PHQ-9 is a self-administered 9-item questionnaire used to assess for the presence of depressive symptoms. The 4-option response format allows scores of 0, 1, 2 and 3 corresponding to “not at all”, “several days”, “more than half the days”, and “nearly every day”, respectively. The total score ranges from 0 to 27, with scores of 5, 10, 15, and 20 representing cut points for mild, moderate, moderately severe, and severe depression, respectively [12]. The PHQ-9 has been validated and extensively used in medical populations, with high sensitivity and specificity both for case detection and diagnosis of depressive disorders. Similarly, the GAD-7 is a self-administered 7-item questionnaire used to assess for the presence of generalized anxiety disorder symptoms. Using the same 4-option responses as the PHQ-9, the total score ranges from 0 to 21, with scores of 5, 10, and 15 representing cut points for mild, moderate and severe anxiety, respectively. The GAD-7 has been validated and extensively used in primary care settings with good sensitivity and specificity to screen for generalized anxiety disorder. Finally, the PHQ-15 is a self-administered 15-item questionnaire used to assess for the presence of symptoms of somatoform disorders in primary care settings. The 3-option response format allows scores of 0, 1, and 2, corresponding to “not at all”, “bothered a little”, and “bothered a lot”. The total score ranges from 0 to 30, with scores of 5, 10, and 15 representing cut points for somatic symptoms of low, medium, and high severity, respectively.

### Procedures

On alternative days during a 5-month period (May 1st to September 30th, 2012), the PHQ-SAD was administered to all the consenting patients seen in the selected primary care clinics, while they waited to be seen by their physicians. The Arabic or the English versions were used as appropriate. Illiterate patients were assisted in completing the questionnaires. Primary care physicians were advised to refer to consulting psychiatrists, all patients who screened positive for a mental disorder.

### Data analyses

The data were analyzed on SSPS, version 22 (IBM corp. Armonk, NY: 2013). Descriptive statistics, including mean, SD, frequencies, and percentages were used to describe the data. Following recommendations in the literature, a cut - off score of 10 or above was used to ascertain the probable prevalence rates of all three mental conditions [13–15]. Thus, somatization, anxiety, and depression total scores were coded as >/= 10 (=1, i.e., clinical depression/ anxiety/ somatization is present) or < 10 (= 0, i.e., no clinical depression/ anxiety/ somatization), and used as grouping variables for chi-square tests with gender, age group, nationality, marital status, and level of education. Similarly, the four physical illnesses were used in chi-square tests with socio-demographic characteristics and mental conditions. To evaluate the impact of multiple physical illnesses, we used one-way ANOVA to assess the association between psychopathology scores and number of physical illnesses. Odds ratio was used for comparing the odds of having a comorbid physical and mental health condition vs. only a physical condition. For all tests, a *p*- value of 0.05 was used to determine statistical significance.

## Results

### Characteristics of the overall sample

Of 1046 participants, aged 18–80 years (mean 37.6, SD 12.7), consisting of 429 males (aged 39.2, SD 12.6), and 617 females (aged 36.5, SD 12.6; *t* = 3.4, df = 1044, *P* < 0.001), 764 (73%) were married, 801 (77%) were Kuwaiti nationals, and 337 (32%) had a University degree.

### Characteristics of the participants with a physical illness

Of the 1046 respondents, 670 (64.1%) had a diagnosis of a physical illness for which at least one medication was prescribed; 383 of them (57.2%) were females, while 287(42.8%) were males; 512 (76.4%) were married; 300 (44.8%) had a College or University degree; and 608 (90.7%) reported their socio-economic status as medium or high (Table 1). Physical illnesses (*N* = 670) included diabetes mellitus (37.0%), hypertension (34.2%), asthma (12.2%), other non-chronic physical illness, such as upper respiratory tract infections or diarrhea (9.4%), and heart disease (7.2%). The mean (SD) age was significantly older in participants with a heart disease (52.7, SD = 13.8 years), hypertension (49.9, SD = 12.6), or diabetes mellitus (49.0, SD = 13.3), than in those with non-chronic illnesses (37.4, SD = 11.3 years) or asthma (35.9, SD = 10.5) (all *p*’s < 0.001).

**Table 1:** Socio-demographic characteristics of participants with physical illnesses (*N* = 670)

Disease	Living condition			Level of education			Marital status				Gender		Age
	n (%)^b^			n (%)			n (%)				n (%)		Mean (SD)
	Low	Medium	High	Illiterate/Primary	High school	College/University	Single	Married	Divor-ced	Widow	Male	Female	
Diabetes (*N* = 248)	28 (11.3)	171 (69.0)	48 (19.8)	98 (39.5)	55 (22.2)	95 (38.3)	16 (6.5)	193 (77.9)	7 (2.8)	32 (12.9)	120 (48.4)	128 (51.6)	49 (13)
Hypertension (*N* = 229)	29 (12.7)	157 (68.6)	43 (18.8)	86 (37.5)	49 (21.4)	94 (42.1)	14 (6.1)	177 (77.3)	6 (2.6)	32 (14.0)	103 (45.0)	126 (55.0)	49.9 (12)
Others^a^ (*N* = 63)	5 (7.9)	45 (71.4)	13 (20.6)	9 (14.2)	13 (20.6)	41 (65.1)	11 (17.5)	46 (73.0)	6 (9.5)	–	15 (23.8)	48 (76.2)	37.4 (11)
Heart disease (*N* = 48)	1 (2.1)	33 (68.8)	14 (29.2)	22 (45.8)	9 (18.8)	17 (35.4)	3 (6.3)	36 (75.0)	1 (2.1)	8 (16.7)	24 (50.0)	24 (50.0)	52.7 (13)
Asthma (*N* = 82)	6 (7.3)	59 (72.0)	17 (20.7)	11 (13.4)	18 (22.0)	53 (64.7)	14 (17.1)	60 (73.2)	6 (7.3)	2 (2.4)	25 (30.5)	57 (69.5)	35.9 (10)

*Other: non-chronic mild conditions, e.g., upper respiratory tract infection, etc

^b^In Kuwait, an affluent country, a total monthly family income for Kuwaiti nationals of <KD 800 (about US$2600), is regarded as low living condition; medium living condition is <KD 2000 (i.e., US$6600); and high living condition is > KD 2000

Of the 1046 participants, 376 (35.9%; i.e., 376/1046) had no diagnosed physical illness, 275 (26.3%) had one, 148 (14.1%) had two, and 33 (3.2%) had 3 or more. In addition to these 456 participants with one or more chronic physical illnesses, 63 had minor non-chronic conditions, and there was insufficient information to categorize the number of comorbid physical illnesses in 151 participants. The number of chronic physical illnesses was not associated with gender, but it was significantly associated with older age (χ ^2^ = 331, df = 9, *p* < 0.0001). Participants who were divorced or widowed were also significantly more likely to have more physical conditions than those who were either single or married (χ ^2^ = 159.5, df = 9, *p* < 0.0001); participants with less than 12 years of education were significantly more likely to have multiple physical illnesses, than those with higher levels of education (χ ^2^ = 152.6, df = 12, *p* < 0.001); and participants with poorer living conditions were significantly more likely to have multiple physical illnesses than those who reported better living conditions (χ ^2^ = 21.2, df = 6, *p* < 0.002).

### Mental - physical comorbidity

Of 1046 participants, 360 (34.4%) had both at least one mental condition and at least one diagnosed physical illness. Hence, the prevalence of a comorbid mental condition was 53.7% (360/670) among those with at least one physical diagnosis, compared to a prevalence of 21.8% (82/376) of at least one mental disorder among those without a diagnosed physical illness. The unadjusted odds ratio (OR) for having a mental condition in those with a physical illness vs. those without a physical illness was 4.16 (95% C.I. = 3.12, 5.55).

Table 2 sought to assess (by chi-square) the association of mental conditions (and their possible combinations) with specific physical illnesses, with a view to seeing whether particular mental conditions occurred more frequently in specific physical conditions. The most common mental condition comorbid with physical illnesses was somatization (17.9–20.8%) or the co-occurrence of all three mental conditions (13.7–28.6%). These associations were statistically significant for hypertension, asthma and non-chronic medical conditions..

**Table 2:** Association of physical and mental conditions

	Diabetes (N = 248)	Hypertension (N = 229)	Others (N = 63)	Heart Disease (N = 48)	Asthma (N = 82)
No Mental Disorder (n, %)	134 (54.0)	110 (48.0)	20 (31.7)	19 (39.6)	27 (32.9)
Somatization (n, %)	34 (13.7)	41 (17.9)	11 (17.5)	10 (20.8)	15 (18.3)
Anxiety (n, %)	3 (1.2)	4 (1.8)	2 (3.26)	0	2 (2.4)
Depression (n, %)	12 (4.8)	6 (2.6)	2 (3.2)	1 (2.1)	2 (2.4)
Somatization + Anxiety (n, %)	10 (4.0)	12 (5.2)	1 (1.6)	3 (6.3)	3 (3.7)
Anxiety + Depression (n, %)	6 (2.4)	7 (3.1)	1 (1.6)	2 (4.2)	2 (2.4)
Somatization + Depression (n, %)	15 (6.0)	16 (7.0)	8 (12.7)	3 (6.3)	13 (15.9)
All 3 mental disorders (n, %)	34 (13.7)	33 (14.4)	18 (28.6)	10 (20.8)	18 (22)
Statistics	χ ^2^ = 6.45	χ ^2^ = 20.7	χ ^2^ = 37.9	χ ^2^ = 13.4	χ ^2^ = 41.3
df	df = 7	df = 7	df = 7	df = 7	df = 7
*p* value	*p* > 0.05	*p* < 0.004	*p* < 0.0001	*p* = 0.063	*p* = 0.0001

The psychopathology scores (i.e., PHQ-9, GAD-7 and PHQ-15 scores) were significantly associated with higher number of physical illnesses (*p* < 0.0001) (see Table 3). Furthermore, as shown in Table 3, although participants with heart diseases or asthma had higher psychopathology scores than participants with diabetes mellitus or hypertension, the differences were statistically significant only for asthma (vs. diabetes and hypertension) and for somatization or depression scores.

**Table 3:** Severity of psychopathology vs. number and types of physical illnesses

Number of physical illnesses^a^	PHQ-15: Somatization	GAD-7: Anxiety	PHQ-9: Depression
	Mean (SD)	Mean (SD)	Mean (SD)
No physical illness (*n* = 376)	6.8 (4.7)	4.6 (4.5)	5.6 (4.9)
One physical illness(*n* = 275)	8.5 (5.9)	5.9 (5.3)	6.8 (6.2)
Two physical illnesses (*n* = 148)	9.6 (6.3)	6.9 (6.2)	8.1 (6.9)
Three or more physical illnesses (*n* = 33)	11.1 (6.2)	8.6 (7.7)	8.7 (6.5)
Statistics, *p* value	F_3, 828_ = 18.9, *p* < 0.001	F_3, 828_ = 13.9, *p* < 0.001	F_3, 828_ = 10.8, *p* < 0.001
Multiple comparisons	3 & 2 illnesses > 1 and no illness	3 & 2 illnesses > 1 and no illness	3, 2, and 1 illnesses > no illness
Specific physical illness			
Diabetes mellitus (n = 248)	8.3 (5.9)	6.3 (6.0)	7.0 (6.6)
Hypertension (n = 229)	8.9 (5.9)	6.5 (6.3)	7.4 (6.6)
Heart disease (n = 48)	10.0 (6.4)	7.5 (7.1)	8.2 (6.8)
Asthma (n = 82)	11.8 (6.6)	7.3 (5.2)	8.9 (6.2)
*p* value	F_3,603_ = 7.4; *p* < 0.01 for asthma vs. diabetes and hypertension	*P* > 0.05	F = 1.9; *p* > 0.05. But for asthma vs. diabetes: *t* = 2.4, *p* < 0.02

^a^In addition to the 376 participants with no physical illness and the 456 participants with one or more chronic physical illnesses, 63 had minor non-chronic conditions, and there was insufficient information to categorize the number of physical illnesses in 151 participants

## Discussion

Using a validated instrument, we assessed the relationship between common mental conditions and comorbid physical illness in a representative sample of Kuwaiti primary care attenders. To our knowledge, this is the first study to assess the prevalence of comorbid mental and physical illness in the Arab World. While depression and anxiety have consistently shown to be more prevalent among women [6], we did not find that comorbid mental and physical disorders were associated with gender. However, they were associated with older age, lower level of education, being divorced or widowhood, and poorer living condition. The association with older age is congruent with the low prevalence of chronic physical illness in younger people [17]. The association with social disadvantage suggests that preventative or therapeutic interventions targeting these social determinants of health could be beneficial and ultimately reduce health care costs [18].

The odds of having a mental condition were more than four-fold higher in those with a physical illness than in those without one. This finding is also congruent with the literature [4, 19–21]. Both the prevalence and the severity of mental conditions increased with the number of comorbid physical illnesses. This “dose-response” relationship identifies a group of primary care attenders at high risk for comorbid mental conditions who could also be the target of specific preventative or therapeutic interventions.

The most common comorbid mental condition in those with a physical illness was somatization, either alone or combined with depression and anxiety. However, there was no robust association between specific mental and physical illnesses. Again, this is congruent with the literature [22]. Despite this lack of specificity, comorbid mental and physical disorders result in poorer prognosis, increased resource utilization, higher health care costs, functional disability, and poorer treatment compliance [4, 21, 23].

The implication of this endorsement of comorbidity as the norm in clinical presentation [24] is that the transdiagnostic approach should be applied in the understanding and treatment of comorbid conditions [25]. This means searching for and treating common underlying phenomena that maintain co-occurring medical conditions, the effective amelioration of which should significantly reduce morbidity [26].

The strengths of this study include the use of a validated instrument in a representative sample. Its limitations include its cross-sectional design and the narrow range of chronic physical illnesses identified in our sample. This narrow range of illnesses seen in a primary care sample is reflective of the health care system in a small affluent country, where patients have easy and direct access to highly specialized hospitals for treating many chronic illnesses such as cancer, neurological, renal, or skin diseases (i.e., these specialist hospitals also offer easy access in walk-in clinics without the need of a referral from primary care physicians). Despite these limitations, our findings are congruent with the literature and support the call for primary care physicians to be mindful of the psychological and social contexts of patients who present primarily with physical illness, particularly if these patients have multiple medical illnesses or are disadvantaged socially.

## Conclusion

Somatization, anxiety, and depression are highly prevalent among primary care attenders in Kuwait, as has been widely reported in the literature, and they are typically comorbid with physical illness. Strategies for their prevention and treatment need to take into consideration their association with physical illness and social disadvantage.

## Abbreviations

ANOVA: Analysis of Variance
CI: Confidence Interval
DSM IV: Diagnostic and Statistical Manual of Mental Disorders 4th Edition
GAD-7: General Anxiety disorder is 7 item questionnaires
PHQ-15: Patient Health Questionnaires is 15 item questionnaires
PHQ-9: Patient Health Questionnaires is 9 item questionnaires
PHQ-SAD: Patient Health Questionnaires for somatization/anxiety/depression
SD: Standard Deviation
SPSS: Statistical Package for Social Science
